# U. S. Dispensatory

**Published:** 1883-02-20

**Authors:** 


### *UNITED STATES DISPENSATORY.*

The last, or eighteenth edition of the United States Dispensatory is reported ready. It has been undergoing revision during the last three years, and is said to embrace all the late discoveries in Pharmacy, Materia Medica and Therapeutics. It will be warmly welcomed by the Profession.

